# A Common *UDP-Glucuronosyltransferase* (*UGT*)*1A* Haplotype Is Associated With Accelerated Aging in Humanized Transgenic Mice

**DOI:** 10.1155/omcl/3203439

**Published:** 2025-03-27

**Authors:** Bettina Langhans, Christian P. Strassburg, Christoph Röcken, Sandra Kalthoff

**Affiliations:** ^1^Department of Internal Medicine I, University Hospital Bonn 53127, Bonn, Germany; ^2^Department of Pathology, University Hospital Schleswig-Holstein 24105, Kiel, Germany

**Keywords:** aging, amyloidosis, cellular senescence, oxidative stress, UDP-glucuronosyltransferases

## Abstract

**Background:** Aging is characterized by the progressive decline of physiological functions and is associated with an increasing risk for developing multiple age-related diseases. UDP-glucuronosyltransferase (UGT)1A enzymes detoxify a variety of endo- and xenobiotic reactive metabolites, thereby acting as indirect antioxidants. A common genetic *UGT1A* haplotype was shown to affect redox balance in humanized transgenic (htg) *UGT1A* mice. Since oxidative stress is a main activator of cellular senescence, we aimed to investigate the role of genetic *UGT1A* variants in the process of aging.

**Methods:** Htg*UGT1A*-WT *and* htg*UGT1A*-SNP mice were harvested at the age of either 12 weeks (young) or 18 months (aged). The effect of aging was examined by analyzing *UGT1A* expression and activity, expression of senescence markers, and senescence-associated secretory phenotype (SASP) factors, as well as blood counts, serum parameter, and histological staining.

**Results:** In comparison to aged htg*UGT1A*-WT mice, hepatic *UGT1A* mRNA and protein expression as well as UGT activity were significantly reduced in aged htg*UGT1A*-SNP mice. Moreover, elderly htg*UGT1A*-SNP mice exhibited increased levels of oxidative stress, senescence markers, SASP factors, and peripheral leukocyte counts compared to the respective htg*UGT1A*-WT mice. Consistent with these findings, we observed higher amounts of collagen and amyloid fibrils as well as an elevated senescence-associated β-galactosidase (SA-β-gal) activity in histological sections of the liver obtained from aged htg*UGT1A*-SNP mice.

**Conclusion:** Our data suggest an accelerated aging process caused by a common *UGT1A* haplotype. Moreover, elderly individuals carrying the *UGT1A* haplotype might exhibit an altered metabolism of drugs, which could necessitate dose adjustments.

## 1. Introduction

Aging is an unavoidable biological process that involves the gradual decline of physiological functions accompanied by an increased risk for the development of life-threatening diseases, such as congestive heart failure, diabetes, renal dysfunction, cancer, and neurodegenerative diseases [1–3]. At the cellular level, cells in various tissues can enter into a permanent cell cycle arrest, which can be induced by different stressors, including oncogene activation, DNA damage, and oxidative stress [4]. These so-called senescent cells cannot proliferate and are resistant to apoptosis but can remain metabolically active for months [5]. Senescent cells exhibit specific morphologic, genetic, and metabolic characteristics, serving as senescence markers, such as the activity of the senescence-associated β-galactosidase (SA-β-gal) [6]. Senescence is also associated with an increased expression of p53, p16^INK4a^, and p21^CIP1^, which enforce the replicative arrest and an impaired expression of numerous genes [7–9]. Moreover, senescent cells can secrete proinflammatory cytokines (e.g., tumor necrosis factor alpha [TNFα], interleukin (IL)-6, andIL-1), chemokines, growth factors, and proteases that affect neighboring cells, a phenomenon known as senescence-associated secretory phenotype (SASP) [10].

Reactive oxygen species (ROS) are considered as one of the main activators of cellular senescence [11, 12]. When ROS concentrations exceed the antioxidant capacity of the cells, oxidative stress occurs, potentially leading to severe damage [13]. The damaging effects of oxidative stress have been associated with aging [14, 15], neurodegenerative diseases, atherosclerosis, heart disease, and cancer [16]. UDP-glucuronosyltransferases (UGT)1As are phase II enzymes and catalyze the formation of glucuronides from a broad array of potentially cytotoxic or genotoxic compounds, which include human carcinogens as well as xeno- and endobiotic reactive metabolites [17–19]. The mechanism of glucuronidation leads to the inactivation of the reactive metabolites; therefore, UGT1As can be described as indirect antioxidants. More than 100 single-nucleotide polymorphisms (SNPs) have been identified within the *UGT1A* coding sequence and the *UGT1A* promoter regions, which can affect protein function as well as transcriptional regulation and have been identified as risk factors for the development of cancer [20–24]. A common *UGT1A* haplotype (UGT1A1*⁣*^*∗*^28, UGT1A3-66 T>C, UGT1A6*⁣*^*∗*^2a, UGT1A7*⁣*^*∗*^3, and UGT1A7-57T>G) is present in 10% of the white population in the homozygous form and is associated with Gilbert syndrome [25]. The genetic *UGT1A* variants included in this haplotype were associated with either low transcription or low enzymatic activity indicating a potential deficit in eliminating reactive metabolites from the organism [25–28]. In previous studies of our laboratory, we were able to show that humanized transgenic (htg) *UGT1A*-SNP mice carrying the *UGT1A* haplotype exhibited higher levels of hepatic and intestinal hydrogen peroxide compared to their wild-type counterparts (htg*UGT1A*-WT mice) [29, 30]. Given that oxidative stress is a major activator of cellular senescence and aging, the low function *UGT1A* haplotype might lead to an accelerated aging process due to the increased levels of oxidative stress. However, to this date, limited data on human *UGT1A* expression in elderly individuals are available. Furthermore, the impact of genetic *UGT1A* variants on the aging process is still unknown. The aim of this study was therefore to examine the expression and activity of UGT1A enzymes in the liver of elderly individuals as well as to characterize the effect of common *UGT1A* polymorphisms on the development of cellular senescence using a htg*UGT1A* mouse model.

## 2. Methods

### 2.1. Compliance with ARRIVE Guidelines

In this study, we utilized a htg*UGT1A* mouse model, which has been used in multiple other studies analyzing the regulation of human *UGT1A* genes [25, 29, 31, 32]. Given their widespread use in metabolic research, mice served as an appropriate model for this study. All experiments were conducted in compliance with the “German Animal Protection Law” and the guidelines of the Local Institutional Animal Care unit of our university (Haus für Experimentelle Therapie, University Hospital Bonn, Germany). Moreover, the study was approved by the relevant North Rhine-Westphalian state agency for Nature, Environment and Consumer Protection (LANUV, Germany).

For our study, untreated male and female htg*UGT1A*-WT and htg*UGT1A*-SNP mice (harboring 10 *UGT1A* SNPs: UGT1A1*⁣*^*∗*^28, UGT1A3-66T>C, UGT1A3 V47A, UGT1A3 W11R, UGT1A6*⁣*^*∗*^2a [S7A/T181A/R184S], and UGT1A7*⁣*^*∗*^3 [N129K/R131K/W208R/-57T>G]) were utilized. At 12 weeks of age, all mice were randomly assigned (block randomization) into test and control groups. The test groups of both mouse lines consisted of each eight male and eight female “aged” mice (total of 32 mice) and were killed untreated after 67 additional weeks of maintenance at the age of 18 months. The control groups of both mouse lines consisted of each eight male and eight female “young” mice (total of 32 mice) and were sacrificed without treatment at the age of 12 weeks.

We housed mice on aspen wood bedding in individually ventilated cages (2–5 mice/cage) and facilitated free access to water and standard irradiated rodent chow (19% protein rodent diet V1534-000, 3.3% fat; Sniff, Soest, Germany). Mice were sacrificed by cervical dislocation. The liver and kidneys were isolated and instantly shock-frozen in liquid nitrogen followed by storing at −80°C. A Vet ABC blood counter (Scil animal care company) was used for blood cell counts. Except for histological analyses, all experimental data collection and analyses were performed without blinding, as the procedures were standardized and automated using preprogrammed routines. This standardized approach minimized operator bias and reduced the necessity for blinding. The histological analyses were performed by a second investigator, which was unaware of the age, gender, or genotype of the analyzed mice.

The sample size for each experimental group was calculated using an online sample size calculator (http://jumbo.uni-muenster.de/fileadmin/jumbo/applets/falla.html). For each analysis, the exact value of number in each experimental group is stated in the respective Methods section. The animals were included in the study if the starting body weights were in the range of 20–25 g (male) or 18–23 g (female). Mice were excluded due to severe illness (referring to a specific score sheet with humane endpoints). No animals, experimental units, or data points were excluded from the study.

The following parameters were assessed: mRNA expression of senescence markers and SASP factors, UGT1A mRNA/protein expression and activity, oxidative stress levels, blood cell counts, serum parameter, and histological staining. The primary endpoint of this study was defined as a significant increase of TNFα expression in htg*UGT1A*-SNP mice compared to htg*UGT1A*-WT mice.

### 2.2. Gene Expression Analysis

RNA was extracted from murine liver using Trizol (Invitrogen, Karlsruhe, Germany) according to the manufacturer's protocol. A total of 5 μg of RNA, extracted from eight individual mice per group, was treated with DNase I (Thermo Fisher Scientific, Schwerte, Germany) for 15 min at room temperature. The reaction was inactivated at 65°C for 10 min. Afterward, complementary DNA (cDNA) was synthesized using DNase I-treated RNA, oligo (dT)-primers, and the SuperScript III First-Strand Synthesis System (Thermo Fisher Scientific, Schwerte, Germany). UGT1A mRNA expression was quantified by qPCR using gene-specific primers and probes and qPCR MasterMix (Eurogentec, Seraing, Belgium). All reactions were performed on a CFX96 real-time PCR detection system (Bio-Rad, Neuried, Germany), with murine beta-actin serving as the reference gene. TaqMan Gene Expression Assays from Thermo Fisher Scientific were used for determination of p65, IL-6, TNFα, tissue growth factor (TGF)β, and IL-1β gene expression analysis (p65, Mm00712720_m1; IL-6, Mm00446190_m1; TNFα, Mm00443260_g1; TGFβ, Mm01178820_m1; and IL-1β, Mm00434228_m1). Quantitative real-time PCR of leucine-rich repeat-containing G protein-coupled receptor (Lgr)5-, p16-, p21-, and p53 was performed using specific primers (Lgr5: forward, 5′GGACCAGATGCGATACCGC, and reverse, 5′CAGAGGCGATGTAGGAGACTG; p16: forward, 5′ATGGAGTCCGCTGCAGACAG, and reverse, 5′ATCGGGGTACGACCGAAAG; p21: forward, 5′ACTTCCTCTGCCCTGCTGC, and reverse, 5′GGTCTGCCTCCGTTTTCG; and p53: forward, 5′CACAGCACATGACGGAGGTC, and reverse, 5′TCCTTCCACCCGGATAAGATG) and Biozym Blue S'Green qPCR Mix (Biozym, Hessisch Oldendorf, Germany). All reactions were performed in three technical replicates and were repeated three times. Relative gene expression was calculated using Bio-Rad CFX Manager 3.0 software.

### 2.3. Western Blot

A pool of liver tissue extracted from eight mice per group (10 mg liver tissue per mouse) was homogenized in 2500 μL RIPA buffer containing proteinase inhibitors followed by incubation at 4°C on a shaker for 1 h. The mixture was then centrifuged (13,000 rpm; 10 min; 4°C), and the supernatant was transferred into new tubes. A total of 40 μg of extracted protein was heated for 5 min (95°C) in Laemmli sample buffer followed by separation by SDS-PAGE and transfer onto a nitrocellulose membrane. Primary antibody incubation with anti-UGT1A (binding to all UGT1A proteins; antibodies-online.com; ABIN2856950) was performed in 10% dry milk. An incubation with suitable secondary antibodies (Millipore, Schwalbach, Germany) followed by detection of protein bands was carried out using chemiluminescence (Biorad, München, Germany) with a ChemiDoc MP imaging system (Bio-Rad). GAPDH staining (anti-GAPDH, Santa Cruz sc-32233) was used as loading control for protein normalization. Three individual experiments for western blot analysis were conducted for each study group.

### 2.4. UGT-Glo Assay

UGT-Glo Assay Kit (Promega, Mannheim, Germany) was used for the evaluation of UGT enzyme activity. In order to isolate microsomes, 10 mg of liver tissue of eight individual mice per group was mechanically disrupted with the help of a QiagenTissue Lyser. The shredded material was suspended in 2.5 mL of buffer (50 mmol/L Tris-HCl [pH 7.4] plus 10 mmol/L MgCl_2_) and further homogenized using a Potter–Elvehjem tissue grinder. The resulting homogenate underwent centrifugation (10,000 × *g*; 5 min; 4°C), and the supernatant was ultracentrifuged at 150,000 × *g* for 60 min at 4°C. Afterward, the final pellet was resuspended in 0.2 mL of buffer. Bradford assay was utilized to determine protein concentrations. Until further analysis, microsomal proteins were stored at −80°C. Each enzymatic reaction was carried out using 1 µg of microsomal protein. Enzyme activity was assessed after a 90-min incubation with 25 µM UGT multienzyme substrate, following the manufacturer's protocol. All experiments were conducted in three individual experiments for each treatment group.

### 2.5. Hydrogen Peroxide Assay

Following the manufacturer's instructions (OxiSelect Hydrogen Peroxide Assay Kit), 10 mg of liver tissue from eight mice per group was homogenized. The measurement of hydrogen peroxide was performed with a Multiskan Go Reader (Thermo Scientific). All experiments were carried out in technical duplicates and three individual experiments for every study group.

### 2.6. Determination of Carbonyl Content

A carbonyl content assay kit (MAK094 Sigma-Aldrich) was used to analyze hepatic protein from eight mice per study group according to the manufacturer's instructions. Samples were measured with a Multiskan Go Reader (Thermo Scientific). All experiments were carried out in technical duplicates and three individual experiments for every study group.

### 2.7. Measurement of Serum Parameter and Blood Cell Counts

For serum analyses, blood was centrifuged at 6000 × *g* for 10 min, and serum was subsequently kept at −20°C until analysis. Triglycerides, creatinine, alanine aminotransferase (ALT), and aspartate aminotransferase (AST) from 16 individual mice per group were determined with a Fuji DRI-CHEM NX500i (Fujifilm Cooperation, Tokyo, Japan) serum analyzer. Blood cell counts (from EDTA blood) were generated using a Vet ABC blood counter (Scil Animal Care Company).

### 2.8. Histological Analyses

Liver/kidney tissue of eight individual mice per group were embedded in paraffin or Tissue-Tek, respectively. To visualize collagen fibers, paraffin-embedded tissue sections were cut into 2.0 µm slices and stained with Sirius red solution (saturated picric acid containing 0.1% DirectRed 80). The proportion of Sirius red–positive area was assessed using ImageJ software (US National Institutes of Health; http://rsb.info.nih.gov/ij/) and expressed as a percentage of the total section area. For each animal, four randomly selected images (magnification 100×) were analyzed, and the mean positive area was calculated. Additionally, hematoxylin and eosin (H&E) staining was performed on 2.0 µm paraffin-embedded tissue sections following a standard protocol to assess histopathological changes. For amyloid staining, Congo Red staining kit (Sigma-Aldrich, Darmstadt, Germany) was used according to the manufacturers protocol. Amyloid deposition was analyzed using polarization microscopy. In our study, the amount of amyloid infiltrating the tissue was divided into four levels (+ = small amount of amyloid infiltrating the tissue [does not include traces]; + + = moderate amount of amyloid infiltrating the tissue; +++ = large amount of amyloid infiltrating the tissue; ++++ = excessive amount of amyloid infiltrating the tissue; *⁣*^*∗*^ = no amyloid was found [traces might have been present]).

Twelve-micrometer-thick cryosections were used for the detection of the SA-β-gal. The staining was performed using the optimized protocol for cryopreserved liver tissue from Jannone et al. [33].

### 2.9. Statistics

Statistical analysis was conducted using Student's *t*-test to compare groups, with significance set at *p* < 0.05.

## 3. Results

### 3.1. *In Vivo* Regulation of UGT1A mRNA Expression in Young and Aged htg*UGT1A* Mice

**Figure 1:**
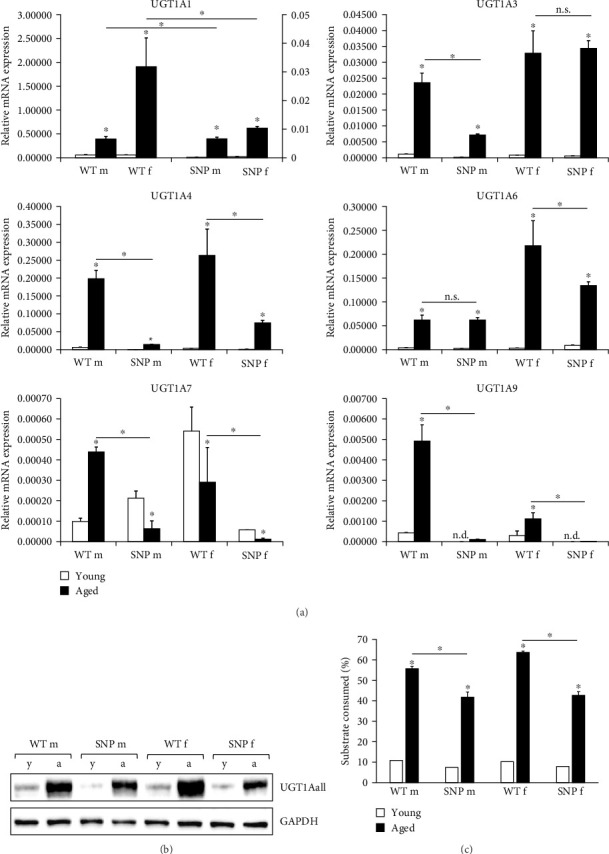
Effects of aging on hepatic UGT1A mRNA and protein expression as well as on UGT activity. (A) UGT1A mRNA expression was highly upregulated in the liver of aged htg*UGT1A*-WT and SNP mice compared to the respective young mice. Columns represent mean ± SD using eight individual mice per group. Significance of aged groups was determined in comparison to the respective young group as asterisk above the bars or as indicated by lines. Values for UGT1A1 mRNA expression in htg*UGT1A*-WT mice refer to the left *y*-axis; UGT1A1 values for htg*UGT1A*-SNP mice refer to the right *y*-axis. (B) Induction of hepatic UGT1A protein expression in aged htg*UGT1A* mice. A representative western blot out of three individual experiments (using each eight mice per group) is shown. (C) Effects of aging on UGT activity. UGT enzyme activity against a proluciferin substrate was determined using UGT-Glo Assay (Promega). UGT activity was significantly induced in the liver of aged htg*UGT1A*-WT and SNP mice. Columns represent mean ± SD using eight individual mice per group. Significance of aged groups was determined in comparison to the respective young group or as indicated by lines. *⁣*^*∗*^*p* < 0.05; a, aged; f, female; m, male; n.s., not significant; y, young.

We first studied hepatic UGT1A mRNA expression in male and female young (12 weeks old) or aged (18 months old) htg*UGT1A*-WT and htg*UGT1A*-SNP mice. Except for UGT1A7, all hepatic UGT1A mRNAs were highly induced in aged htg*UGT1A* mice compared to their young counterparts (Figure 1A). UGT1A1 mRNA was significantly upregulated in male (sevenfold) and female (33-fold) aged htg*UGT1A*-WT mice. In the liver of htg*UGT1A*-SNP mice, UGT1A1 mRNA expression was 41-fold increased in old males and 27-fold in females compared to young SNP mice. However, absolute expression levels of old htg*UGT1A*-SNP mice remained far below levels measured in the respective WT mice (64-fold lower in aged SNP males compared to aged WT males and 184-fold lower in old SNP females compared to old WT females). UGT1A3, UGT1A4, UGT1A6, and UGT1A9 mRNA levels were significantly induced in the liver of aged htg*UGT1A*-WT and SNP mice in comparison to young mice. However, despite some exceptions (UGT1A3 in females and UGT1A6 in males), the expression of the mentioned *UGT1A*s was significantly reduced in old SNP mice compared to aged htg*UGT1A*-WT mice. In contrast to all other contemplated *UGT1A*s, *UGT1A7* was significantly downregulated in aged htg*UGT1A*-SNP mice and old WT females. Only male aged htg*UGT1A*-WT mice exhibited a significant *UGT1A7* upregulation compared to the respective young mice. In line with the expression of other *UGT1A*s, *UGT1A7* expression in aged htg*UGT1A*-SNP mice was significantly lower than in old htg*UGT1A*-WT mice, independent of gender.

In summary, these data suggest that the aging process can significantly increase mRNA expression of most UGT1A enzymes in the liver of both htg*UGT1A*-WT and SNP mice. However, htg*UGT1A*-SNP mice exhibit significant lower expression levels in comparison to WT mice.

### 3.2. *In Vivo* Regulation of Hepatic UGT1A Protein Expression in Young and Aged htg*UGT1A* Mice

Using an antibody binding to all UGT1A isoforms (anti-UGT1Aall) in Western blot analysis, we determined UGT1A protein levels in the liver. Compared to young htg*UGT1A* mice, aged htg*UGT1A*-WT and SNP mice showed highly elevated UGT1A protein expression in the liver (Figure 1B). However, protein levels in young and aged htg*UGT1A*-SNP mice were reduced compared to the respective WT mice.

### 3.3. Effect of Aging on UGT Enzyme Activity in the Liver of htg*UGT1A* Mice

In order to investigate whether UGT enzyme activity is increased in the liver of aged htg*UGT1A* mice, we measured UGT activity using UGT-Glo Assay (Promega). As shown in Figure 1C, UGT activity was highly induced in aged htg*UGT1A*-WT and SNP mice compared to young mice. Furthermore, the activity levels in old htg*UGT1A*-SNP mice were significantly reduced in comparison to aged WT mice, confirming the findings from the analysis of UGT1A mRNA and protein expression. Therefore, these data indicate a lower glucuronidation capacity in aged htg*UGT1A*-SNP mice compared to their WT counterparts.

### 3.4. Increased Levels of Oxidative Stress in the Liver of Aged htg*UGT1A* Mice

**Figure 2:**
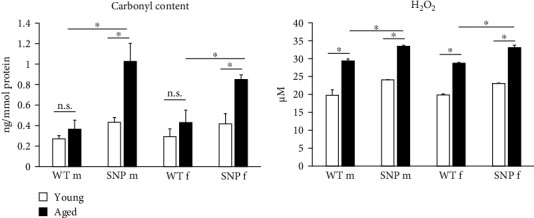
Effects of aging on hydrogen peroxide levels and carbonyl content in the liver of htg*UGT1A*-WT and SNP mice. Carbonyl content and hydrogen peroxide levels were significantly higher in old htg*UGT1A*-SNP mice compared to aged WT mice. Significance was determined as indicated by lines. Columns represent mean ± SD using eight individual mice per group. *⁣*^*∗*^*p* < 0.05; f, female; m, male; n.s., not significant.

Increased oxidative stress is assumed to be associated with the process of aging. Oxidative stress can cause post-translational modifications of proteins including carbonylation. Protein carbonylation, a detrimental and irreversible protein modification caused by oxidative stress, is recognized as a key indicator of oxidative stress. Therefore, we sought to examine the carbonyl content as well as hydrogen peroxide levels in the liver of young and aged htg*UGT1A*-WT and SNP mice. In contrast to htg*UGT1A*-WT mice, we observed a significant increase of carbonyl content in aged htg*UGT1A*-SNP mice compared to young counterparts (Figure 2). Moreover, the carbonyl content was significant higher in old htg*UGT1A*-SNP mice than in aged WT mice. In line with this data, hepatic hydrogen peroxide levels were significantly elevated in both aged htg*UGT1A*-WT and SNP mice. However, H_2_O_2_ levels were significantly higher in old SNP mice compared to old htg*UGT1A*-WT mice.

### 3.5. *In Vivo* Expression of Hepatic Senescence Markers in Young and Aged htg*UGT1A* Mice

**Figure 3:**
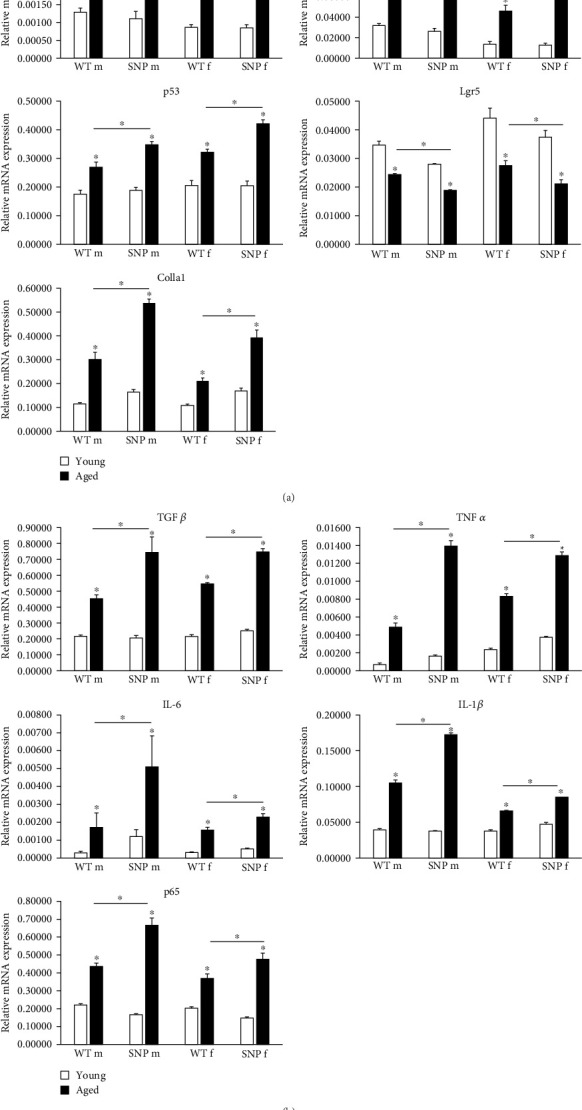
Expression of senescence markers and SASP factors in the liver of young and aged htg*UGT1A* mice. (A) mRNA expression of p16^INK4a^, p21^CIP1^, p53, and Col1a1 was significantly upregulated in aged mice, although significant higher expression levels were observed in old htg*UGT1A*-SNP mice compared to old WT mice. In comparison to aged htg*UGT1A*-WT mice, aged SNP mice exhibited a significantly reduced expression of Lgr5 mRNA. (B) SASP factors TGFβ, TNFα, IL-6, and IL-1β and SASP-regulating factor p65 were significantly higher transcribed in aged htg*UGT1A*-SNP mice compared to old htg*UGT1A*-WT mice. Columns represent mean ± SD using eight individual mice per group. Significance of aged groups was determined in comparison to the respective young group or as indicated by lines. *⁣*^*∗*^*p* < 0.05; f, female; m, male; n.s., not significant.

Transcription of the aging-associated senescence markers p16^INK4a^ and p21^CIP1^ was significantly increased in the liver of all aged htg*UGT1A* mice, although the expression levels were significantly higher in htg*UGT1A*-SNP mice than in WT mice (Figure 3A). p53 plays a crucial role in cellular senescence by regulating numerous target genes responsible for cell cycle arrest. Similar to p16^INK4a^ and p21^CIP1^, hepatic p53 mRNA expression was significantly higher in aged htg*UGT1A*-SNP mice in comparison to aged WT mice. Lgr5 is a biomarker of adult stem cells and involved in the Wnt signaling pathway. Lgr5 generally promotes proliferation and was shown to be downregulated during the process of aging [34]. Consistently, Lgr5 mRNA expression was significantly decreased in aged livers of both mouse lines. However, the reduction of *Lgr5* transcription was more pronounced in old htg*UGT1A*-SNP mice compared to old WT mice. In the study of Loobma et al. [35], it was shown that accelerated aging is associated with a higher hepatic collagen content in patients with nonalcoholic steatohepatitis (NASH), and Maeso-Diaz et al. [36] found increased Col1a1 expression levels in the liver of aged rats. In consistence with these findings, we observed an upregulated expression of Col1a1 mRNA in aged htg*UGT1A* mice, which was significantly higher in htg*UGT1A*-SNP mice. In summary, these data indicate an accelerated aging process in the liver of aged htg*UGT1A*-SNP mice.

### 3.6. Aging Leads to Increased Expression of SASP Factors and SASP-Regulating Factor p65 in htg*UGT1A* Mice

Senescent cells produce and release a variety of proinflammatory cyto- and chemokines, proteases, and growth factors, known as SASP. SASP is a potential driver of age-related dysfunction and has a profound effect on the surrounding cells. In aged htg*UGT1A* mice, we observed a significant transcriptional upregulation of the SASP factors TGFβ, TNFα, IL-6, and IL-1β (Figure 3B). In comparison to aged htg*UGT1A*-WT mice, mRNA expression levels of the analyzed SASP factors were significantly elevated in aged htg*UGT1A*-SNP mice. p65 was shown to be a master regulator of SASP [37, 38]. In our study, p65 transcription was significantly upregulated during aging in the liver of htg*UGT1A* mice. Similar to the examined SASP factors, p65 expression levels were significantly higher in aged htg*UGT1A*-SNP mice compared to the respective WT mice.

### 3.7. Aging Leads to Increased Number of Leukocytes in Aged htg*UGT1A*-SNP Mice

**Figure 4:**
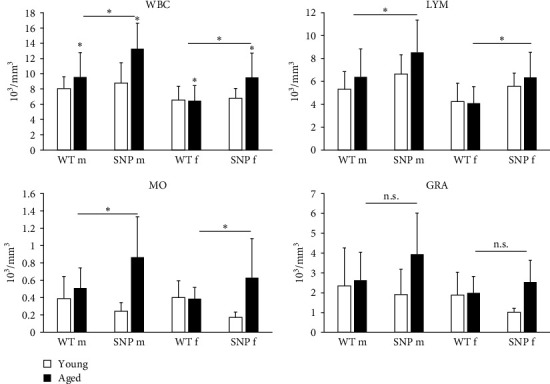
Effect of aging on WBC, lymphocytes, monocytes, and granulocytes in htg*UGT1A*-WT and SNP mice. WBC, lymphocytes, monocytes, and granulocytes were significantly higher in aged htg*UGT1A*-SNP mice than in aged WT mice. Columns represent mean ± SD using 16 individual mice per group. Significance of aged groups was determined in comparison to the respective young group as asterisk above the bars or as indicated by lines. GRA, granulocytes; LYM, lymphocytes; MO, monocytes. *⁣*^*∗*^*p* < 0.05; f, female; m, male; n.s., not significant.

As reliable indicators of inflammation, increased counts of white blood cells (WBCs) together with elevated expression of proinflammatory cytokines are related to various chronic diseases. Therefore, we compared leukocyte numbers between young and aged htg*UGT1A* mice. Except for aged female htg*UGT1A*-WT mice, leukocyte number was significantly increased in old mice compared to young counterparts (Figure 4). In agreement with our data on increased expression of cytokines in aged htg*UGT1A*-SNP mice, we observed significantly higher numbers of leukocytes in old SNP mice than in old htg*UGT1A*-WT mice. Similarly, the numbers of lymphocytes and monocytes were significantly higher in aged htg*UGT1A*-SNP mice compared to the respective WT mice. Although there was also a trend to higher amounts of granulocytes in aged htg*UGT1A*-SNP mice, the difference was statistically not significant.

### 3.8. Effect of Aging on Hepatic Collagen Deposition and Aminotransferase Activities

**Figure 5:**
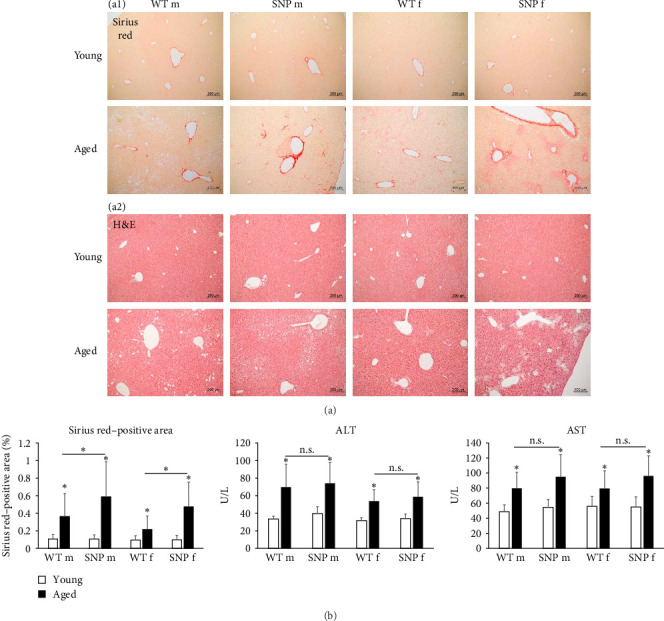
Effect of aging on liver histology and serum aminotransferases. Representative hepatic sections of histological Sirius red staining (A) ((a1) young versus aged) or H&E staining (A) ((a2) young versus aged) in htg*UGT1A* mice (magnification 100×). (B) Computational analysis of Sirius red–positive areas and determination of aspartate aminotransferase (AST) and alanine aminotransferase (ALT) activities in serum of young and aged htg*UGT1A* mice. Significantly increased levels of Sirius red–positive areas were observed in aged htg*UGT1A*-SNP mice compared to aged WT mice. Columns represent mean ± SD using eight individual mice per group for histological analysis and 16 mice per group for the detection of AST and ALT. Significance of aged groups was determined in comparison to the respective young group or as indicated by lines. *⁣*^*∗*^*p* < 0.05; f, female; m, male; n.s., not significant.

Since aging leads to increased collagen deposition in the liver and we observed a transcriptional increase in hepatic Col1a1 expression, liver sections were stained with Sirius red. As expected, histological evaluation revealed elevated levels of red-stained collagen fibers in aged mice compared to young mice (Figure 5A). Of note, computational analysis revealed a significant higher amount of Sirius red–positive areas in aged htg*UGT1A*-SNP mice in comparison to aged WT mice (Figure 5B). Although statistically not significant, serum aminotransferases AST and ALT levels were also slightly higher in aged htg*UGT1A*-SNP mice compared to aged WT mice (Figure 5B). Strikingly, we detected various acellular eosinophilic deposits in the stroma and around vessels in H&E-stained liver sections of female aged htg*UGT1A*-WT and SNP mice (Figure 5A). In aged males, we only observed these eosinophilic deposits in one htg*UGT1A*-WT and in one htg*UGT1A*-SNP mouse. We assumed that the detected deposits could indicate amyloid deposition; therefore, we proceeded with Congo Red Staining.

### 3.9. Hepatic Amyloid Deposition and SA-β-Gal Activity in Aged htg*UGT1A*-WT and SNP Mice

**Figure 6:**
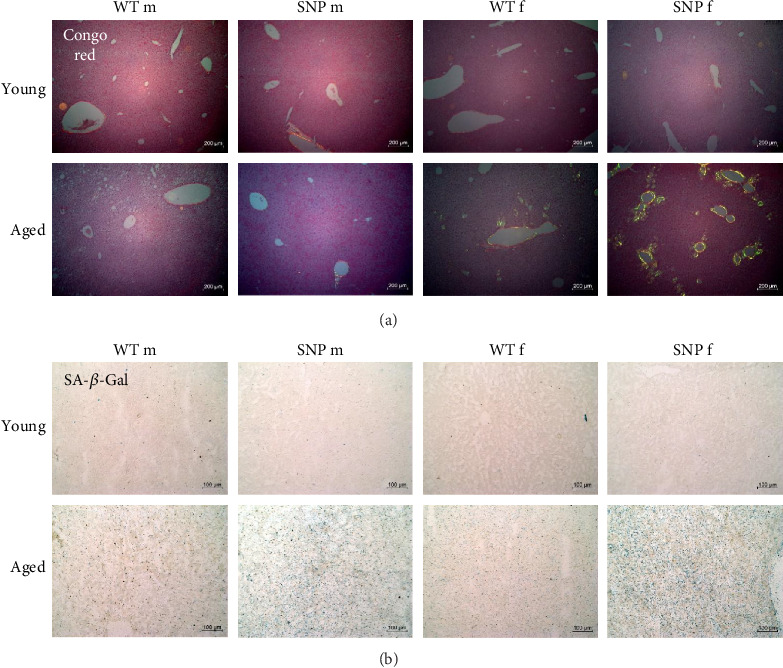
Amyloid deposition and SA-β-gal activity in the liver of young and aged htg*UGT1A* mice. Representative hepatic sections of histological Congo Red staining from eight individual mice per group (A) (young versus aged, magnification 100×) or SA-β-gal staining (B) (young versus aged; polarized microscopy, magnification 200×) in htg*UGT1A* mice. Higher amounts of amyloid deposits were observed in aged female htg*UGT1A*-SNP mice compared to female WT counterparts. SA-β-gal activity was significantly elevated in aged male and female htg*UGT1A*-SNP mice compared to aged WT mice. f, female; m, male.

To obtain evidence for amyloid deposition, liver sections were stained with Congo Red and analyzed using polarization microscopy. As expected, we were not able to find significant accumulation of amyloid in the liver of young mice (Figure 6). In contrast, aged female htg*UGT1A*-WT and SNP mice exhibited typical yellow to apple-green birefringence in polarized light. We observed hepatic amyloid deposition in 50% of aged female htg*UGT1A*-WT mice, whereas amyloid deposits were present in 87.5% of aged female htg*UGT1A*-SNP mice (Table 1). In addition to a higher prevalence, the extend of amyloid deposition was significantly higher in aged female htg*UGT1A*-SNP mice compared to their aged female WT counterparts. In contrast, we only counted one amyloid-positive old male htg*UGT1A*-WT mouse and one old male SNP mouse, respectively.

To obtain direct evidence of cellular senescence in aged mice, we examined the presence of SA-β-gal in the liver. As shown in Figure 6 (bottom), we observed an accumulation of SA-β-gal activity in aged livers, whereas this was not evident in young livers. Interestingly, we detected a profoundly higher SA-β-gal activity in the liver of aged htg*UGT1A*-SNP mice compared to aged WT mice. Furthermore, SA-β-gal staining was more intense in aged female htg*UGT1A*-SNP mice than in old SNP males.

In summary, aging leads to a much more pronounced deposition of amyloid in the liver of aged female htg*UGT1A*-SNP mice compared to old female WT mice, whereas males exhibited nearly no amyloid deposits. In comparison to aged htg*UGT1A*-WT mice, hepatic SA-β-gal activity was higher in both male and female aged htg*UGT1A*-SNP mice.

### 3.10. Renal Amyloid Deposition and Serum Creatinine Levels in Aged htg*UGT1A*-WT and SNP Mice

**Figure 7:**
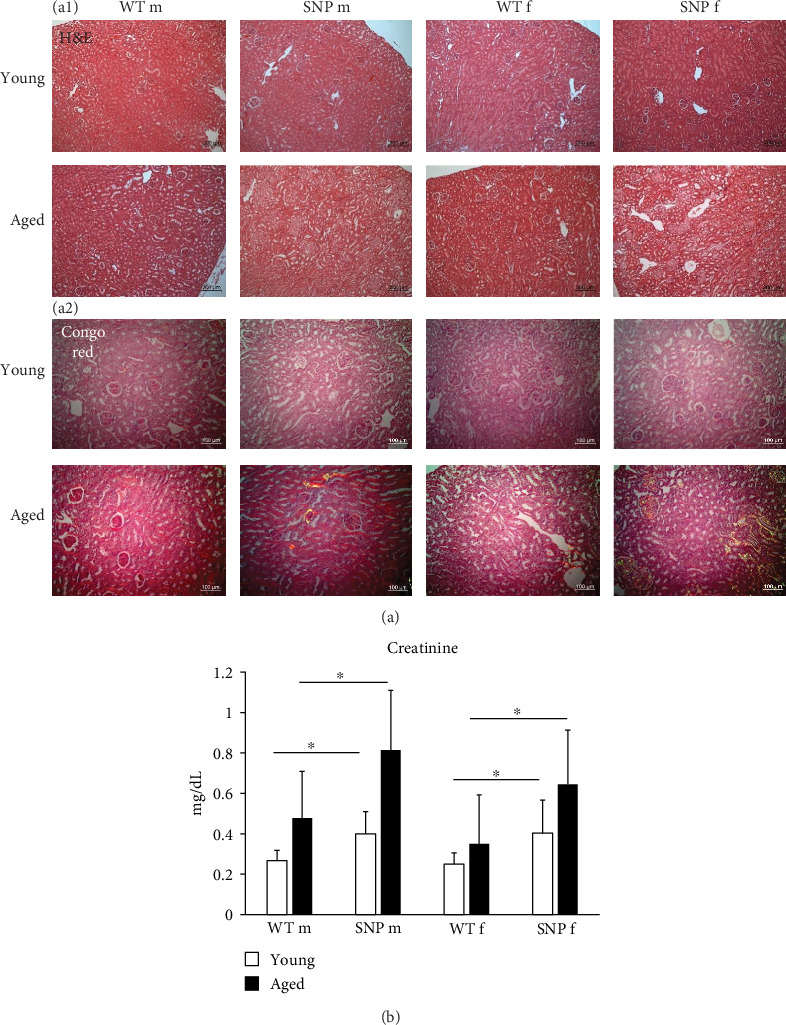
H&E staining and amyloid deposition in the kidney of young and aged htg*UGT1A* mice. Representative renal sections of histological H&E staining from eight individual mice per group (A) ((a1) young versus aged, magnification 100×) or Congo Red staining (A) ((a2) young versus aged; polarized microscopy, magnification 200×) in htg*UGT1A* mice. (B) Creatinine levels were significantly higher in the serum of htg*UGT1A*-SNP mice than in WT mice. Columns represent mean ± SD using 16 individual mice per group. Significance was determined as indicated by lines. *⁣*^*∗*^*p* < 0.05; f, female; m, male; n.s., not significant.

The liver and the kidney are functionally closely intertwined. Both organs play an essential role in the elimination of metabolic waste products and toxins. Liver diseases often coexist with renal dysfunctions. Therefore, we also histologically examined the kidneys. Similar to the results obtained from liver histology, we found areas with acellular eosinophilic deposits in some H&E stained sections from aged mice (Figure 7A (a1)), which again were shown to be amyloid in Congo Red-stained sections. Similar to the liver, we observed amyloid in renal tissue, especially in aged female htg*UGT1A*-SNP mice (Figure 7A (a2) bottom and Table 2). The prevalence for amyloid was 0% for aged male htg*UGT1A*-WT mice, 25% for aged male htg*UGT1A*-SNP mice, 38% for aged female htg*UGT1A*-WT mice, and 50% for aged female htg*UGT1A*-SNP mice. However, aged female htg*UGT1A*-SNP mice had the largest amount of amyloid in the kidney. Interestingly, serum creatinine levels were also significantly increased in htg*UGT1A*-SNP mice compared to their WT counterparts.

## 4. Discussion

The liver is the primary organ for metabolizing both endogenic and xenobiotic compounds. Age-related functional changes in the liver can contribute to increased susceptibility to age-related diseases [39]. During the process of aging, the expression and activity of hepatic enzymes involved in biotransformation may undergo significant alterations. Many cytochrome P450 (CYP) enzymes exhibit low transcription levels during fetal and neonatal stages, rise as the liver matures, and decrease substantially in old age, as observed in aged rats [40].

There are several studies showing an absence of human *UGT1A* expression in fetal livers and a progressive increase in expression between neonates, young children, and adults [41–44]. However, data concerning human *UGT1A* expression in elderly individuals are not available, and data concerning UGT activity during aging are inconsistent. Vyskocilova et al. [45] showed, for example, that Ugt activity was significantly decreased in old rats compared to young rats using *p*-nitrophenol as a substrate. Two other studies found Ugt activity to be unaffected by aging in rats [46, 47]. Here, we demonstrated that human hepatic UGT1A mRNA expression, protein expression, and activity were significantly increased by aging in humanized tg*UGT1A* mice. The profound age-related upregulation of *UGT1A* expression might represent a compensatory mechanism to adjust to emergent higher amounts of reactive metabolites in aged tissue. The differences found across several studies investigating the effect of aging on UGT enzymes may be caused by analyzing human UGT enzymes versus rodent Ugts and by potential inconsistence between substrates used for examining UGT activity. Moreover, to our knowledge, there are no data available showing mRNA and protein expression of human UGT1A enzymes in aged individuals apart from this study. Remarkably, *UGT1A* expression and activity levels of aged htg*UGT1A*-SNP mice remained significantly below those detected in aged htg*UGT1A*-WT mice. These data are in line with results obtained from other studies of our laboratory, where we could show that the presence of the *UGT1A* haplotype leads to a significant reduction of *UGT1A* expression and inducibility under different conditions [25, 29, 30, 48]. Although our data show that the hepatic *UGT1A* expression in aged htg*UGT1A*-SNP mice is lower than in their aged WT counterparts, the overall *UGT1A* expression in both aged groups is significantly higher than in the respective young mice. Clinically, this suggests that elderly individuals—regardless of *UGT1A* genotype—may require higher doses of drugs metabolized via glucuronidation. However, in those with *UGT1A* haplotype, careful consideration should be given to both the reduced function of the UGT1A enzymes due to the genetic variants and the potential compensatory higher expression levels observed with aging. Further research is necessary analyzing the efficacy and safety of medications in elderly individuals with known *UGT1A* genotype to refine dosing guidelines.

The accumulation of ROS during aging leads to a profound level of oxidative stress. Several studies have indicated a connection between oxidative stress and cellular injury in elderly individuals [49, 50]. We observed significantly elevated levels of carbonyl content and hydrogen peroxide in the liver of aged htg*UGT1A* mice. Interestingly, oxidative stress measured in htg*UGT1A*-SNP mice was significantly higher than in the respective WT mice. This observation is in line with data from our previous studies showing higher levels of hydrogen peroxide in irinotecan or benzo (a)pyrene treated htg*UGT1A*-SNP mice compared to htg*UGT1A*-WT mice [29, 30]. Probably due to a reduced glucurondation capacity toward reactive metabolites, htg*UGT1A*-SNP mice exhibit a larger accumulation of ROS in the liver, which might constitute a risk factor for an accelerated aging process.

As the main activator of senescence, imbalanced ROS can lead to DNA damage and activated p53 and p16^INK4a^. p53 in turn activates its transcriptional target p21^CIP1^, thereby promoting cell cycle arrest [51]. In our experiments, we also observed increased expression of the senescence markers p16^INK4a^, p21^CIP1^, and p53 in the liver of elderly htg*UGT1A* mice, whereby aged htg*UGT1A*-SNP mice showed significantly higher expression in comparison to the respective WT mice. Similarly, Col1a1 mRNA expression as well as the expression of different SASP factors were significantly elevated in the liver of elderly htg*UGT1A*-SNP mice compared to their aged WT counterparts. Moderate fibrosis was shown to be a histological hallmark of the aging liver [52, 53]. Consistent with this finding, we observed in addition to the transcriptional upregulation of Col1a1 expression also an increase of Sirius red–positive tissue areas of aged livers from htg*UGT1A* mice. The presence of the *UGT1A* haplotype in aged htg*UGT1A*-SNP mice triggered a more pronounced collagen deposition in comparison to the respective WT mice. These data indicate that the liver of aged SNP mice displays a higher degree of collagen deposition, inflammation, and senescence compared to the respective htg*UGT1A*-WT mice, suggesting an accelerated aging process in the liver of SNP mice. Significant higher numbers of WBCs, lymphocytes, and monocytes in blood counts also suggest an increased degree of inflammation in aged htg*UGT1A*-SNP mice.

Although serum ALT levels decrease with age in humans, several studies have shown an increase of serum ALT and AST in aged mice [54, 55]. In this study, aging also led to an elevation of serum ALT and AST levels in htg*UGT1A* mice. ALT and AST levels were also slightly higher in aged htg*UGT1A*-SNP mice compared to aged WT mice, but this trend was statistically not significant. Further evidence for accelerated aging in htg*UGT1A*-SNP mice was attained by the analysis of hepatic SA-β-gal activity. A significant higher SA-β-gal activity was detected in aged htg*UGT1A*-SNP mice compared to the respective WT mice.

Amyloidosis encompasses a group of protein-folding disorders marked by the extracellular accumulation of insoluble amyloid fibrils resulting from abnormal conformational changes [56]. The deposition of amyloid fibrils is a key factor in the development of age-related diseases, including Parkinson's and Alzheimer's disease as well as wild-type transthyretin amyloidosis (ATTRwt). The incidence of ATTRwt rises with age reaching a peak incidence in the eight decades of life and can impact multiple organs, including the liver, the spleen, the heart, kidneys, nervous system, and digestive tract [57]. Aging-related amyloidosis also affects mice and is caused by deposition of apolipoprotein A-II (murine AApoAII amyloidosis) [58]. In the liver, we observed a significant deposition of amyloid fibrils in female aged htg*UGT1A* mice, whereby the prevalence and severity of amyloid accumulation was more profound in the presence of the *UGT1A* haplotype. For some types of amyloidosis (e.g., human ATTRwt and murine AApoAII), aging is known as a main risk factor [57]. Therefore, the higher levels of oxidative stress, senescence, and inflammation measured in female htg*UGT1A*-SNP mice might lead to a more severe deposition of amyloid compared to female htg*UGT1A*-WT mice. Interestingly, amyloid accumulation was nearly absent in the liver of male mice indicating gender differences. Although SA-β-gal activity was slightly higher in females, most of the aging-related parameters analyzed in this study were not significantly higher in females. Therefore, the reason for the predominant occurrence of hepatic deposition in females remains to be clarified and further studies are required. Renal amyloid deposits were more common in females, but here, the difference between htg*UGT1A*-WT and SNP mice was more profound than the difference between genders. Large amounts of renal amyloid deposits were especially detected in aged female *UGT1A*-SNP mice and in one male htg*UGT1A*-SNP mouse. Serum creatinine levels were also higher in aged htg*UGT1A*-SNP mice compared to the respective WT mice, which is in line with other studies showing that systemic amyloidosis often affects the kidney including elevated serum creatinine [59]. Murine AApoAII amyloidosis affects both the liver and the kidney [60]. In order to confirm this amyloid type in aged htg*UGT1A* mice, further studies are required.

The effects of the *UGT1A* haplotype on aging suggest potential therapeutic interventions to reduce oxidative stress and compensate for *UGT1A* deficiency. Our previous studies identified coffee as a strong *UGT1A* inducer [31], significantly mitigating disease severity in both htg*UGT1A*-WT and htg*UGT1A*-SNP mice across various disease models suggesting a robust protective effect even in the presence of the *UGT1A* haplotype [30, 48, 61]. We further identified caffeic acid (CA) and caffeic acid phenylethyl ester (CAPE) as key mediators of this effect. Thus, CA and CAPE may serve as promising pharmacological candidates to enhance UGT1A activity and counteract the impact of the *UGT1A* haplotype in aging populations.

There may be some possible limitations in this study. Murine models do not fully replicate human aging processes, since aging is a multifactorial process influenced by the interplay of genetic, environmental, and physiological factors. While our htg*UGT1A* mouse model provides a controlled system to isolate the impact of human *UGT1A* gene variants, it does not fully replicate the complex interactions and environmental exposures encountered during human aging. Moreover, our mouse model is only partially humanized and thus may result in species-specific interactions that may not accurately reflect human biology.

## 5. Conclusions

In conclusion, this is the first study showing human UGT1A mRNA and protein expression in elderly individuals using a htg *UGT1A* mouse model. Beside a general upregulation of *UGT1A* expression in aged animals, htg*UGT1A*-SNP mice exhibited a significantly reduced *UGT1A* expression compared to the respective htg*UGT1A*-WT mice. Probably due to a reduced glucuronidation capacity, higher levels of oxidative stress, senescence, inflammation, and amyloid deposition (in females) were observed in aged htg*UGT1A*-SNP mice, indicating an accelerated aging process in the presence of the *UGT1A* haplotype. Moreover, we suppose that elderly individuals carrying the *UGT1A* haplotype might exhibited an altered metabolism of drugs, which could necessitate dose adjustments of medication.

## Figures and Tables

**Table 1:** Liver: Amount of amyloid infiltrating tissue in aged htg*UGT1A* mice.

Mouse line	Sex	ID	Amyloid amount	Mouse line	Sex	ID	Amyloid amount
htg*UGT1A*-WT	m	1	*⁣* ^*∗*^	htg*UGT1A*-SNP	m	9	*⁣* ^*∗*^
htg*UGT1A*-WT	m	2	*⁣* ^*∗*^	htg*UGT1A*-SNP	m	10	*⁣* ^*∗*^
htg*UGT1A*-WT	m	3	+	htg*UGT1A*-SNP	m	11	*⁣* ^*∗*^
htg*UGT1A*-WT	m	4	*⁣* ^*∗*^	htg*UGT1A*-SNP	m	12	*⁣* ^*∗*^
htg*UGT1A*-WT	m	5	*⁣* ^*∗*^	htg*UGT1A*-SNP	m	13	+
htg*UGT1A*-WT	m	6	*⁣* ^*∗*^	htg*UGT1A*-SNP	m	14	*⁣* ^*∗*^
htg*UGT1A*-WT	m	7	*⁣* ^*∗*^	htg*UGT1A*-SNP	m	15	*⁣* ^*∗*^
htg*UGT1A*-WT	m	8	*⁣* ^*∗*^	htg*UGT1A*-SNP	m	16	*⁣* ^*∗*^
htg*UGT1A*-WT	f	17	*⁣* ^*∗*^	htg*UGT1A*-SNP	f	25	+++
htg*UGT1A*-WT	f	18	+	htg*UGT1A*-SNP	f	26	+++
htg*UGT1A*-WT	f	19	+++	htg*UGT1A*-SNP	f	27	++++
htg*UGT1A*-WT	f	20	++	htg*UGT1A*-SNP	f	28	+++
htg*UGT1A*-WT	f	21	*⁣* ^*∗*^	htg*UGT1A*-SNP	f	29	*⁣* ^*∗*^
htg*UGT1A*-WT	f	22	*⁣* ^*∗*^	htg*UGT1A*-SNP	f	30	++
htg*UGT1A*-WT	f	23	*⁣* ^*∗*^	htg*UGT1A*-SNP	f	31	+
htg*UGT1A*-WT	f	24	+	htg*UGT1A*-SNP	f	32	++

*Note:* The amount of amyloid infiltrating liver tissue was divided into four levels (+ = small amount of amyloid infiltrating the tissue [does not include traces]; ++ = moderate amount of amyloid infiltrating the tissue; +++ = large amount of amyloid infiltrating the tissue; ++++ = excessive amount of amyloid infiltrating the tissue; *⁣*^*∗*^ = no amyloid was found [traces might have been present]).

**Table 2:** Kidney: Amount of amyloid infiltrating tissue in aged htg*UGT1A* mice.

Mouse line	Sex	ID	Amyloid amount	Mouse line	Sex	ID	Amyloid amount
htg*UGT1A*-WT	m	32	*⁣* ^*∗*^	htg*UGT1A*-SNP	m	40	*⁣* ^*∗*^
htg*UGT1A*-WT	m	33	*⁣* ^*∗*^	htg*UGT1A*-SNP	m	41	*⁣* ^*∗*^
htg*UGT1A*-WT	m	34	*⁣* ^*∗*^	htg*UGT1A*-SNP	m	42	*⁣* ^*∗*^
htg*UGT1A*-WT	m	35	*⁣* ^*∗*^	htg*UGT1A*-SNP	m	43	+
htg*UGT1A*-WT	m	36	*⁣* ^*∗*^	htg*UGT1A*-SNP	m	44	+++
htg*UGT1A*-WT	m	37	*⁣* ^*∗*^	htg*UGT1A*-SNP	m	45	*⁣* ^*∗*^
htg*UGT1A*-WT	m	38	*⁣* ^*∗*^	htg*UGT1A*-SNP	m	46	*⁣* ^*∗*^
htg*UGT1A*-WT	m	39	*⁣* ^*∗*^	htg*UGT1A*-SNP	m	47	*⁣* ^*∗*^
htg*UGT1A*-WT	f	48	*⁣* ^*∗*^	htg*UGT1A*-SNP	f	55	*⁣* ^*∗*^
htg*UGT1A*-WT	f	49	+	htg*UGT1A*-SNP	f	56	*⁣* ^*∗*^
htg*UGT1A*-WT	f	50	+	htg*UGT1A*-SNP	f	57	*⁣* ^*∗*^
htg*UGT1A*-WT	f	51	*⁣* ^*∗*^	htg*UGT1A*-SNP	f	58	+++
htg*UGT1A*-WT	f	52	*⁣* ^*∗*^	htg*UGT1A*-SNP	f	59	++
htg*UGT1A*-WT	f	53	*⁣* ^*∗*^	htg*UGT1A*-SNP	f	60	*⁣* ^*∗*^
htg*UGT1A*-WT	f	54	*⁣* ^*∗*^	htg*UGT1A*-SNP	f	61	++
htg*UGT1A*-WT	f	55	+	htg*UGT1A*-SNP	f	62	++

*Note:* The amount of amyloid infiltrating kidney tissue was divided into four levels (+ = small amount of amyloid infiltrating the tissue [does not include traces]; ++ = moderate amount of amyloid infiltrating the tissue; +++ = large amount of amyloid infiltrating the tissue; ++++ = excessive amount of amyloid infiltrating the tissue; *⁣*^*∗*^ = no amyloid was found [traces might have been present]).

## Data Availability

The data that support the findings of this study are available from the corresponding author upon reasonable request.
